# The benefit of intraperitoneal chemotherapy for the treatment of colorectal carcinomatosis

**DOI:** 10.3892/or.2013.2473

**Published:** 2013-05-15

**Authors:** VALERIE FRANCESCUTTI, LOUIS RIVERA, MUKUND SESHADRI, MINHYUNG KIM, MICHELLE HASLINGER, MARTA CAMORIANO, KRISTOPHER ATTWOOD, JOHN M. KANE, JOSEPH J. SKITZKI

**Affiliations:** 1 Department of Surgical Oncology, Roswell Park Cancer Institute, Buffalo, NY 14263, USA; 2 Department of Pharmacology and Therapeutics, Roswell Park Cancer Institute, Buffalo, NY 14263, USA; 3 Department of Immunology, Roswell Park Cancer Institute, Buffalo, NY 14263, USA; 4 Department of Biostatistics and Bioinformatics, Roswell Park Cancer Institute, Buffalo, NY 14263, USA

**Keywords:** hyperthermic intraperitoneal chemoperfusion, chemotherapy, intraperitoneal, carcinomatosis, mouse

## Abstract

The clinical practice of hyperthermic intraperitoneal chemoperfusion (HIPEC) for carcinomatosis has lacked preclinical justification. A standardized mouse model was created to evaluate the independent effects of intraperitoneal chemotherapy. Diffuse colorectal carcinomatosis was generated in mice prior to intraperitoneal lavage with mitomycin C (MMC) at clinically comparable dosing for variable lengths of time. Tumor volumes, MMC tissue concentrations and survival were measured in comparison to saline lavage and intravenous MMC. Magnetic resonance imaging revealed a direct correlation between tumor volume, MMC dose and exposure time and survival. Intravenous MMC demonstrated a rapid clearance from the blood, lower peritoneal tissue concentrations, less tumor growth inhibition and decreased survival compared to intraperitoneal administration. Intraperitoneal chemotherapy inhibited tumor growth independent of cytoreduction or hyperthermia, demonstrated improved peritoneal tissue concentration and was associated with increased survival. These data support the clinical utility of the intraperitoneal chemotherapy component of HIPEC.

## Introduction

Colorectal cancer is the third most common cancer in the United States with a significant risk of recurrence by peritoneal seeding following potentially curative surgery (1,2). In general, the survival for patients with colorectal carcinomatosis is poor (3). Traditional systemic chemotherapy has a limited potential for survival prolongation or cure. The 2-year survival rate for patients with colorectal carcinomatosis treated with systemic chemotherapy alone is a dismal 10% (4).

The peritoneum may be the only site of metastatic disease in ~25% of recurrent colorectal cancer patients (1). Therefore, improved survival or even ‘cure’ might be achieved by eradicating disease from all peritoneal surfaces (5). As a regional therapy, removal of all visible tumor deposits within the peritoneal cavity, termed cytoreduction, has been associated with improved survival (6,7). Clinically, this may also require the stripping of parietal and/or visceral peritoneum and solid organ resections (8). As an adjunct to cytoreduction, the intraoperative administration of hyperthermic, high-dose chemotherapy to the peritoneal cavity is thought to eliminate microscopic residual disease. The best studied chemotherapy agent used for hyperthermic intraperitoneal chemoperfusion (HIPEC) is mitomycin C (MMC), however, other agents have also been employed (9,10).

A randomized phase III clinical trial has shown that long-term survival for colorectal cancer patients undergoing cytoreduction/HIPEC is significantly improved as compared to systemic chemotherapy alone, 20 vs. 5%, respectively, at 6 years (7) and cytoreduction/HIPEC has continually gained acceptance within the oncology community. Interestingly, the concept of cytoreduction/HIPEC was immediately used for clinical patient care; it was not developed, translated, or refined from an animal model.

Animal models, especially murine models, present an attractive modality to study and test future therapies for humans. A recent rat model of cytoreduction/HIPEC has been developed that allows for surgical cytoreduction and treatment with intraperitoneal MMC (11). A study using this rat model showed that the addition of HIPEC to cytoreduction improved survival. However, the central issue of completeness of cytoreduction in animal models, similar to cytoreduction/HIPEC in humans, may be a confounding variable, making the study of the independent effects of intraperitoneal chemotherapy difficult. Taking a reductionist approach, we developed a mouse model that mimics the post-cytoreductive state and allows for the contributions of intraperitoneal chemotherapy to be examined in the absence of cytoreduction and hyperthermia. Using this model, the dose and exposure time of intraperitoneal chemotherapy appeared to significantly influence the clinical outcome in a beneficial manner.

## Materials and methods

### Mice

Age-matched BALB/c female mice (10 weeks of age) were purchased from the National Cancer Institute (Frederick, MD, USA). Mice were maintained in pathogen-free barrier conditions. The Roswell Park Cancer Institutional Animal Care and Use Committee approved all animal protocols.

### Tumor cell line

The murine tumor cell line colon 26 (CT26), a weakly immunogenic colon cancer cell line derived from BALB/c mice, was obtained from the American Type Culture Collection (ATCC, Rockville, MD, USA). Cells were maintained in a monolayer culture with RPMI-1640 media (Gibco, Invitrogen Inc., Grand Island, NY, USA) containing 10% fetal bovine serum, 2 mmol/l L-glutamine, 0.15% sodium bicarbonate and 1% penicillin/streptomycin at 37°C in a humidified atmosphere containing 5% CO_2_.

### Establishment of carcinomatosis model

Establishment of diffuse carcinomatosis was performed by injecting BALB/c mice intraperitoneally with 5×10^4^ live CT26 cells in 2 ml of buffered saline using a 27-gauge needle and 6-ml syringe. Necropsy was performed at different time points to determine the optimal time point for intraperitoneal chemotherapy that would mimic the post-cytoreductive microscopic disease-state. Serosal and mesenteric tumor implantation was assessed and digitally photographed with the aid of a dissecting microscope (VWR International, Radnor, PA, USA) at ×8–10 magnification. Frozen section of representative day 2 tumor implant was performed with hematoxylin solution (Sigma-Aldrich, St. Louis, MO, USA) counterstain. All mice underwent intravenous or intraperitoneal treatment 2 days after tumor injection to allow for tumor dissemination and implantation onto the peritoneal surfaces.

### Chemotherapy

Mitomycin C (MMC; Santa Cruz Biotechnology, Inc., Santa Cruz, CA, USA) was suspended in sterile normal saline immediately prior to use at concentrations of 6 μg/ml (low dose) and 8 μg/ml (high dose), corresponding to 15 and 20 μg per mouse, respectively. Total volume administered intraperitoneally was 2.5 ml. For intravenous administration, a more concentrated solution of 15 or 20 μg in 200 μl of sterile saline was used. All solutions were kept at room temperature.

### Intravenous chemotherapy administration

To simulate systemic chemotherapy administration provided clinically, a single bolus of MMC was provided via tail vein injection using a 29-gauge needle. Low dose was provided as 15 μg in 200 μl sterile saline, and high dose at 20 μg in 200 μl sterile saline, which was equivalent to the total low and high dose provided intraperitoneally.

### Mouse intraperitoneal chemotherapy

**Figure 1:**
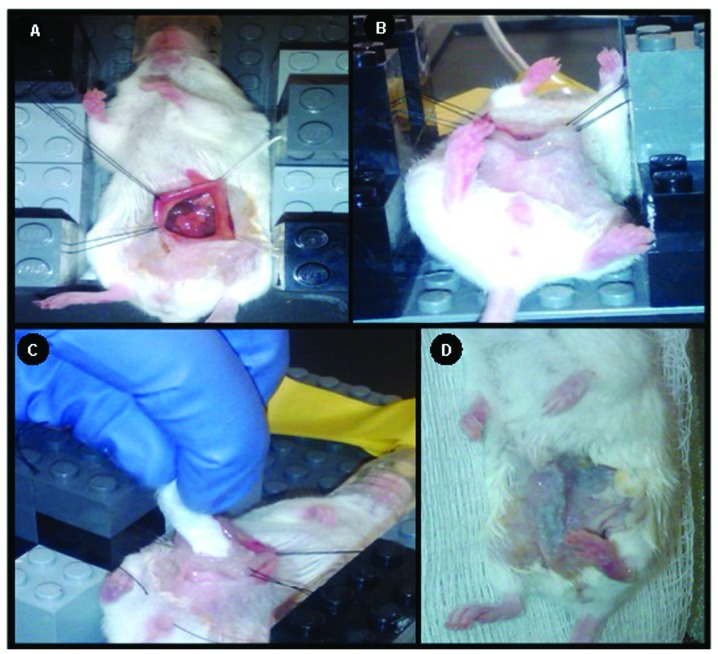
(A) Coliseum technique with tenting of peritoneum by securing silk sutures to Lego^®^ platform; (B) creation of a petroleum jelly rim to allow complete filling of the abdominal cavity; (C) post-treatment, wicking of chemoperfusate from abdominal cavity using sterile 4 × 4 gauze; (D) abdominal closure with absorbable suture and Vetbond™.

Induction of anesthesia was achieved by inhalation of 4% isofluorane (Abbott Laboratories, Chicago, IL, USA) in a sealed chamber. Mice were then placed on a Lego^®^ (Billund, Denmark) platform directly over a water circulating heating pad to maintain normothermia. Anesthesia was maintained during intraperitoneal chemotherapy through controlled regulation of ~2% isofluorane by means of an inlet tube and individual anesthetic nose-cones. The abdominal wall was prepped by clipping overlying hair and applying alcohol to the skin. The abdominal wall and peritoneal cavity were opened with scissors and retracted in a fixed, coliseum fashion using 4-0 Vicryl^®^ suture (Ethicon, Inc., Somerville, NJ, USA) anchored to the platform constructed from building blocks (Fig. 1A). A layer of petroleum jelly was applied to the retracted skin edges to create a barrier to prevent perfusate loss and ensure chemotherapy contact with all peritoneal surfaces (Fig. 1B). Exactly 2.5 ml of MMC or vehicle control (saline) was pipetted into the peritoneal cavity and distributed intraperitoneally for either 60 or 90 min with constant agitation on a programmable shaker (160 rpm) at room temperature. All solutions were evacuated prior to closure of the abdomen by wicking the perfusate with sterile 4 × 4 gauze and washing with sterile saline (Fig. 1C). The peritoneum was closed with a 4-0 absorbable monofilament suture and the skin with Vetbond™ Tissue Adhesive (3M Company, St. Paul, MN, USA) (Fig. 1D). All mice were recovered on a warming blanket and given buprenorphine (0.2 mg/kg body weight) subcutaneously for post-operative pain control.

### In vitro assays of tumor cell viability

CT26 cells were cultured in 25-cm flasks (Costar, Corning Inc., Corning, NY, USA) to 3×10^6^ cell density in 5 ml of media. To confirm the cytotoxic activity of the MMC doses used *in vivo*, 1 ml of high dose MMC (8 μg/ml) was added to individual flasks. Cell death was assessed after the addition of MMC by light microscopy, and dead cells were identified by their non-adherence and cellular debris.

### Magnetic resonance imaging

Preclinical magnetic resonance imaging (MRI) examinations were carried out using a 4.7 T/33-cm horizontal bore magnet (GE NMR Instruments, Fremont, CA, USA) incorporating Avance digital electronics (Bruker BioSpec, ParaVision 3.1.; Bruker Medical, Billerica, MA, USA), a removable gradient coil insert (G060; Bruker Medical) generating a maximum field strength of 950 mT/m, and a custom designed 35-mm radiofrequency transmit-receive coil. Anesthetized animals (achieved by 2–3% isoflurane inhalation) were placed in a form-fitted MR-compatible ‘mouse sled’ (Dazai Research Instruments, Toronto, Canada) within a carrier tube and positioned in the scanner. The body temperature of animals during image acquisition was maintained using an air heater system (SA Instruments Inc., Stony Brook, NY, USA) connected to a thermocouple embedded within the sled that provided feedback for automated temperature control. Animals were imaged 21 days post intraperitoneal inoculation of CT26 tumor cells (19 days post laparotomy/intraperitoneal chemotherapy).

Preliminary localizer images were acquired to enable positioning of slices for T2-weighted scans. T2-weighted spin echo images incorporating RARE (rapid acquisition with relaxation enhancement) encoding were acquired on the coronal plane for each mouse using the following parameters: TE/TR = 41/2500 msec, slice thickness 1 mm, 21 slices, field-of-view (FOV): 4.8 × 3.2 cm (coronal). Post processing of datasets was performed using the medical imaging software, Analyze PC (Version 8.0; AnalyzeDirect, Overland Park, KS, USA). A region of interest was manually traced over the total extent of the tumor on the coronal T2-weighted image. Tumor volume was calculated by measuring the cross-sectional area on each slice and multiplying their sum by the slice thickness. Values are reported as means ± standard error for each experimental group.

### Survival measurements

The mice were followed until tumor growth caused a moribund status and/or any of the criteria for humane euthanasia per approved protocols were observed. Survival was assessed and recorded as the time to euthanasia.

### Whole blood and peritoneum concentrations of MMC

To determine the distribution of MMC in the mouse, administered via tail vein bolus injection vs. peritoneal administration, ultra-high performance liquid chromatography (UPLC) was used. Mice underwent tail vein injection of 20 μg MMC in 200 μl sterile saline or treatment with 20 μg (8 μg/ml) MMC intraperitoneally. For mice treated intraperitoneally, chemotherapy solution was wicked out and irrigated with sterile saline prior to peritoneum specimen collection. Intraperitoneal treatment with MMC occurred to a maximum of 90 min. After this time point, wicking and washout occurred and mice at the 120- and 180-min time points did not have any further intraperitoneal chemotherapy administered. At time points 5, 15, 30, 60, 90, 120 and 180 min, mice were sacrificed and whole blood was collected via 2.5-ml collection tubes containing EDTA (Greiner Bio-One, Monroe, NC, USA) and the entire parietal peritoneum surgically dissected and placed into 15-ml conical tubes (BD Biosciences, San Jose, CA, USA) for immediate freezing.

### Sample preparation for UPLC

Whole blood samples were thawed and centrifuged briefly to pull sample to the bottom of the tube. Samples were sonicated for 1 min in a Branson 2510 sonicator water bath (Branson Ultrasonic Corp., Danbury, CT, USA). Sonicated samples were vortexed and extracted using 200 μl aliquots. Peritoneum samples were pre-weighed and homogenized in 1 ml 75% 0.01 M NaH_2_PO_4_ pH 7.0:25% MeOH using a Polytron PT 2100 homogenizer (Kinematica, Inc., Bohemia, NY, USA). Homogenized samples were extracted using 200 μl aliquots.

### Extraction method

Samples (200 μl) were extracted with 1 ml of HPLC grade acetonitrile (VWR), vortexed for 1 min and centrifuged at 14,000 rpm for 15 min at 4°C. Supernatants (1.1 ml) were transferred to 13 × 100 mm glass tubes and dried under nitrogen at 37°C. Samples were reconstituted in 80 μl of 10 mM NaH_2_PO_4_ at pH 7.0 and vortexed for 15 sec twice. Contents were transferred to microcentrifuge tubes and centrifuged at 14,000 rpm for 15 min at 4°C. Supernatants were transferred to a 0.45 μm 96-well filter plate (VWR) and centrifuged at 3,400 rpm for 6 min at 4°C. Filtrates were transferred to amber autosampler vials for injection.

### UPLC conditions

Samples (40 μl) were injected onto a 100 × 2.1 mm internal diameter Acquity UPLC BEH C18 column, particle size 1.7 μm (Waters, Milford, MA, USA) with a column temperature of 40°C. Mitomycin C was eluted by a two-solvent gradient using a Waters Acquity UPLC system. Solvent A contained 0.01 M NaH_2_PO_4_, pH 7.0 buffer. Solvent B contained HPLC grade MeOH (VWR). The gradient began at 0.2 ml/min with a 4 min hold at 75% solvent A. At 4 min the flow rate changed to 0.3 ml/min and progressed linearly to 10% solvent A over 1 min. Column was held at 10% solvent A for 5 min before returning to initial conditions. Mitomycin C was detected at a UV wavelength of 365 nm. Data were collected and analyzed using Waters Empower Pro chromatography software (version 6.21; Waters).

### Statistical analysis

Tumor volume was reported as the mean ± one standard deviation for each group. The association between tumor volume and group was assessed using a one-way ANOVA test, with post-hoc pair-wise comparisons conducted using Scheffe’s test. The assumptions of the ANOVA test were assessed graphically and indicated the need for a log transformation. Survival data were analyzed using standard Kaplan-Meier methods, with comparisons made using the log-rank test. All analyses were conducted in SAS v9.3 (SAS Institute Inc., Cary, NC, USA) at a nominal significance level of 0.05. All experiments were performed in triplicate to verify reproducibility and tumor volume and survival data were pooled for analyses.

## Results

### Intraperitoneal injection of CT26 mimics human colorectal carcinomatosis

**Figure 2:**
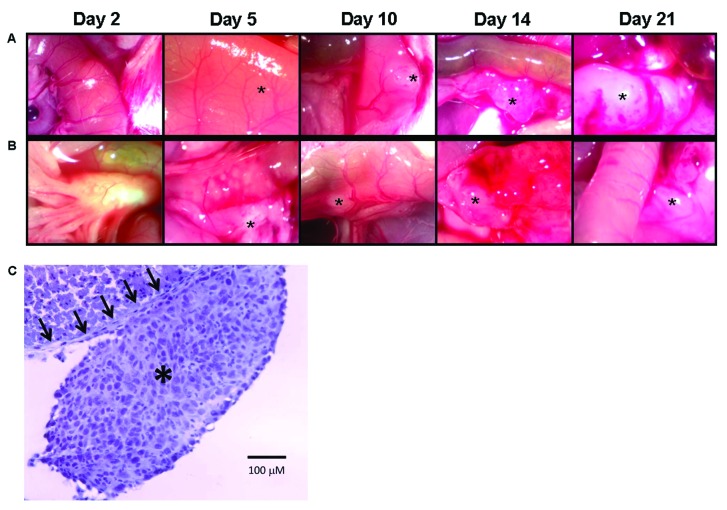
Necropsy images 2, 5, 10, 14 or 21 days after intraperitoneal injection of 5×10^4^ live CT26 cells, showing consistent and progressive (A) small bowel serosal disease and (B) mesenteric disease (magnification, ×8–10) indicated by an asterisk. (C) Frozen section of peritoneum on day 2 after tumor injection demonstrates microscopic tumor implant (asterisk) on the surface of the peritoneum defined by arrows.

To determine the optimal time for the establishment of microscopic intraperitoneal tumor similar to the clinical post-cytoreductive state, necropsy was performed on mice at various time points following CT26 tumor inoculation. Tumor implants became visible (indicated by asterisk on the images) within 5 days of tumor injection both on the small bowel serosa (Fig. 2A) and small bowel mesentery (Fig. 2B). Carcinomatosis developed in 100% of the mice and was progressive resulting in rapid dissemination and growth on all peritoneal surfaces. If left untreated, 100% mortality was noted by day 30. On day 2 following tumor injection, no visible lesions were noted, but diffuse microscopic nodules were present on the peritoneal surfaces (Fig. 2C) and this time point was chosen as it best represented microscopic disease similar to the clinical post-cytoreductive state.

### Murine MMC dosing based upon clinical HIPEC protocols is well tolerated and efficacious

Confirming the *in vivo* antitumor effects and potency of the MMC used for intraperitoneal chemotherapy, verification of MMC activity *in vitro* demonstrated 100% cell death between 36 and 120 h for high-dose and low-dose MMC, respectively. Mice underwent intravenous bolus injection or intraperitoneal administration of chemotherapy with either low dose (6 μg/ml; 15 μg/mouse) or high dose MMC (8 μg/ml; 20 μg/mouse) based upon a MMC/body weight ratio equivalent to the clinical dose of 30 mg of MMC used during HIPEC for a 70 kg individual (the average mouse weight ranged from 25 to 35 g). The standard dose reduction seen in clinical HIPEC from 40 to 30 mg is reflected in the murine doses tested. Mice tolerated the high or low dose MMC and the short or long-duration treatments equally well and did not demonstrate any perioperative morbidity or mortality.

**Figure 3:**
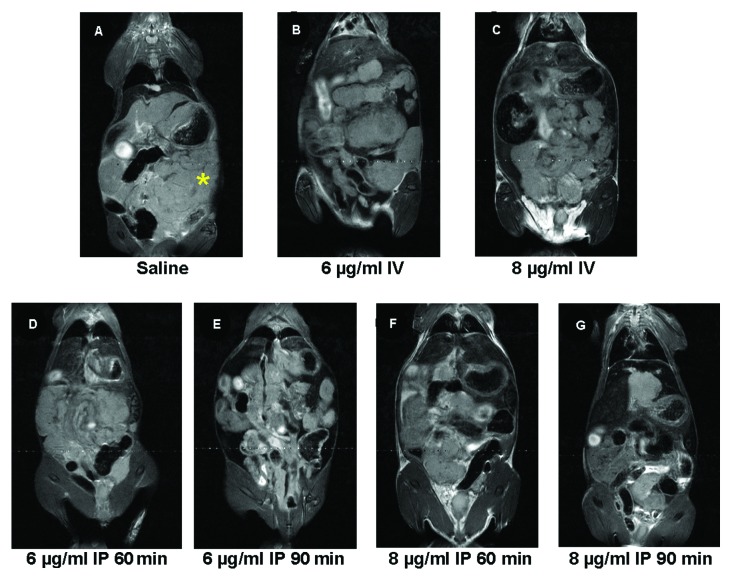
The panel of images represent a single slice from coronal T2-weighted MR images for an animal in each experimental group including saline controls. A diffuse pattern of tumor growth in the peritoneum was visualized on the MR images of saline treated control animals with a reduced pattern in the treatment groups. Peritoneal carcinomatosis was well visualized as MRI offered exceptional soft tissue contrast that allowed for accurate, non-invasive volumetric assessments.

**Figure 4:**
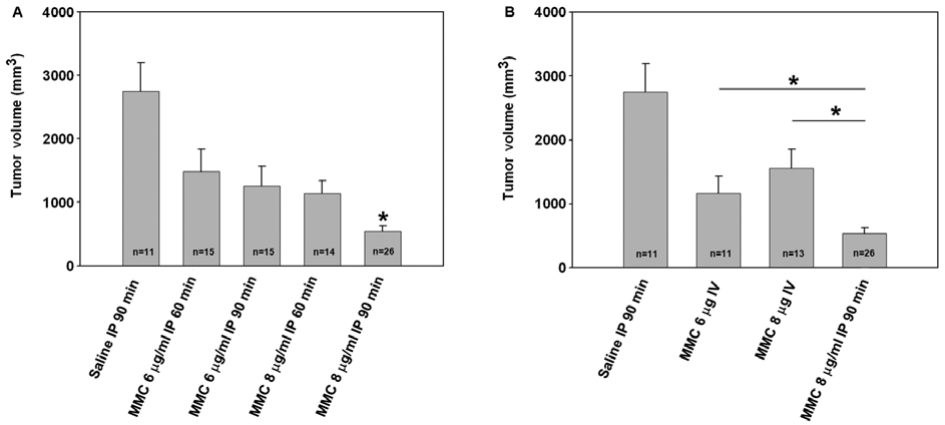
(A) Calculated tumor volumes from MR imaging shows a trend toward tumor inhibition associated with increasing intraperitoneal MMC dosing and exposure time compared to saline controls. Mice treated by high dose MMC given for 90 min had a statistically significant decrease in tumor volumes compared to saline controls (P<0.001). (B) Intravenous MMC treated mice trended toward decreased tumor growth compared to saline controls, but a superior antitumor effect is seen when MMC is given intraperitoneally compared to either intravenous treatments (P<0.05).

The panel of images shown in Fig. 3 represents slices from coronal T2-weighted MRI for all experimental groups. Corresponding tumor volume measurements are shown in Fig. 4. A diffuse pattern of tumor growth in the peritoneum was visualized on the MR images mimicking clinical colorectal carcinomatosis (Fig. 3). Quantitative estimates of tumor volume demonstrated a time and dose-dependent intraperitoneal chemotherapeutic effect. Extensive tumor-burden was visualized in saline-treated control animals (2748±446 mm^3^, n=11) on day 21 post tumor cell inoculation. Mice treated with low dose MMC (for 60 or 90 min) showed a trend to a reduction in tumor volume compared to saline controls (60 min; 1480±352 mm^3^, n=15; 90 min; 1252±318 mm^3^, n=15). Treatment with high dose MMC for 60 min resulted in a comparable trend to reduction in tumor volume (1141±193 mm^3^, n=14). Intravenous MMC at low or high dose also resulted in a non-statistically significant reduction in tumor burden compared to controls. However, the greatest reduction in tumor burden following treatment was seen with high dose MMC for 90 min (538±89 mm^3^, n=26) which was statistically significant as compared to controls (Fig. 4A, P<0.001) and intravenous MMC treated mice (Fig. 4B, P<0.05).

### Tissue concentrations and rate of clearance of MMC vary by the route of delivery

**Figure 5:**
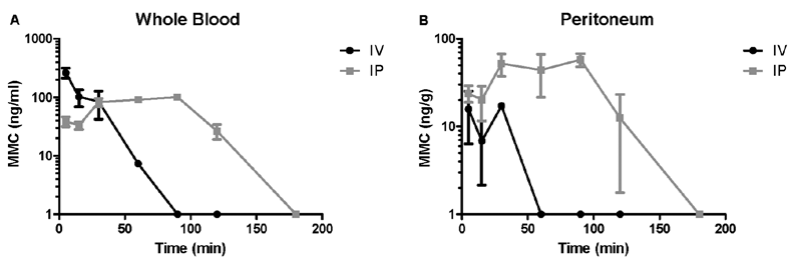
(A) Whole blood UPLC analysis demonstrates a rapid peak and clearance in MMC concentrations when given intravenously vs. a rapid, but prolonged presence following intraperitoneal delivery. (B) Parietal peritoneum UPLC analysis for MMC shows a similar pattern of rapid peak concentration and quick clearance when given intravenously. Peritoneal MMC concentrations quickly peak and are maintained for a prolonged period even after washout of the peritoneum at 90 min (tissue concentrations presented in logarithmic scale).

Tissue distribution of MMC after intravenous or intraperitoneal administration, with respect to whole blood or parietal peritoneum was determined by UPLC. In the whole blood, an immediate peak in MMC and a gradual decline to below detectable limits by 90 min was observed for the intravenous groups. In contrast, the whole blood samples of intraperitoneally administered MMC peaked with a plateau from 30 to 90 min, and gradual decline extending to 180 min (Fig. 5A and B). Peritoneal tissue concentrations of intravenously administered MMC demonstrated a peak from 5 to 30 min with a steep decline that reached levels below quantification at 60 min. On the contrary, intraperitoneal administration of MMC resulted in a plateau in the peritoneal tissue from 30 to 90 min with a slow decline to 180 min (Fig. 5A and B). Overall, intravenous administration led to a rapid peak and subsequently quick elimination of MMC in the tissues studied, whereas intraperitoneal administration led to a more prolonged whole blood concentration and a higher peritoneal concentration that was persistent from 30 to 120 min. It should be noted that the intraperitoneal chemotherapy was evacuated from the peritoneum and the peritoneal cavity lavaged with saline at the completion of 60 or 90 min, similar to clinical protocols, yet, peritoneal tissue concentrations of MMC persisted well beyond this point of evacuation.

### Intraperitoneal treatments associated with lower tumor burden also demonstrate improved survival

**Figure 6:**
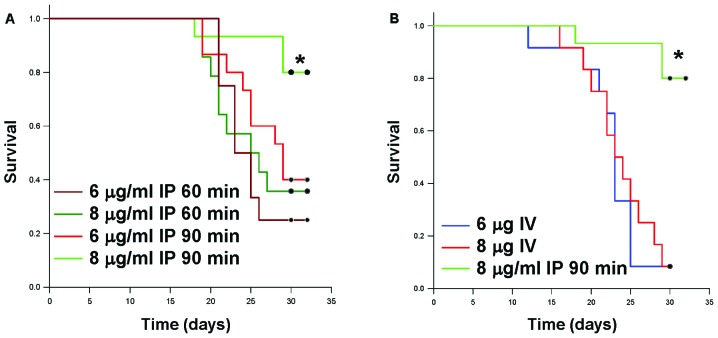
Survival is exposure time- and dose-dependent in mice receiving intraperitoneal chemotherapy lavage. (A) Intraperitoneal MMC (8 μg/ml) given for 90 min exhibited a greater improvement in survival compared to both 6 μg/ml for 90 min and 8 μg/ml for 60 min (^*^P<0.05). (B) Intravenous MMC-treated mice had decreased survival compared to standard HIPEC protocol conditions (i.e. 8 μg/ml intraperitoneal MMC given for 90 min) (^*^P<0.01).

Survival appeared to be correlated to the tumor burden results, as the high dose intraperitoneal MMC-treated mice had improved survival compared with low dose intraperitoneal MMC treatment, intravenous treatment or saline control. Mice receiving a lower dose of MMC (6 μg/ml IP) or mice receiving decreased exposure time to the MMC lavage (i.e. 60 min), had a significantly decreased survival (Fig. 6A). In agreement with the tumor growth inhibition results, mice receiving intravenous MMC had a significantly decreased survival as compared to the optimal intraperitoneal treatment (Fig. 6B). Overall, the majority of mice in the saline, intravenous, and low dose intraperitoneal MMC groups died or met the criteria for euthanasia between 25 and 30 days post-implantation, while the mice treated with high dose MMC for 90 min demonstrated improved survival.

## Discussion

The overall objective of the present study was to examine the effects of varying the dose and exposure time of intraperitoneal chemotherapy on treatment outcome in a clinically relevant animal model. The present study was designed to eliminate the variability of both cytoreduction and hyperthermia in order to focus solely on the contribution of the intraperitoneal chemotherapy component of HIPEC. The model is unique as it replicated the prolonged anesthesia, laparotomy, intraperitoneal chemotherapy dwell times with agitation, and chemotherapy wash out performed clinically in patients. Importantly, mice subjected to the intraperitoneal chemotherapy component of HIPEC in this fashion tolerated the procedure well with no perioperative morbidity or mortality. The dosing range of MMC in the present study corresponded with the clinically accepted dose used for human HIPEC and demonstrated significant time- and dose-dependent tumor growth inhibition. The high dose of MMC for 90 min displayed the greatest tumor growth inhibition and was statistically associated with improved survival. These results indicate that the intraperitoneal chemotherapy component of HIPEC contributes to the observed clinical outcomes independent of cytoreduction and hyperthermia. Furthermore, intraperitoneal administration of MMC was shown in the present study, through UPLC, to result in an overall higher peritoneal concentration of chemotherapy, longer tissue retention time, and a more prolonged whole blood level, which may explain the improved tumor control and prolonged survival seen with the intraperitoneally treated mice.

A potential survival benefit and reduction in tumor volume associated with intraperitoneal chemotherapy has been reported in other murine models (11–13). However, many of these preclinical studies have administered a single intraperitoneal injection of chemotherapy agent without prolonged anesthesia, laparotomy, or the washout that occurs during clinical HIPEC (12,14,15). In studies that attempt to mimic clinical HIPEC, the variability in cytoreduction (11,16,17) or the method of tumor establishment (13,17) may be confounding variables. The mouse model used for the currently reported study differs from other models in that the tumor volume was constant and recapitulated the microscopic disease burden that remains after the completion of cytoreduction. To determine tumor volumes, previous studies have utilized necropsy evaluation or imaging methods such as PET in HIPEC models (11,13,18). In the present study, assessment of intraperitoneal tumor burden was performed non-invasively using MRI, given its ability to provide high soft tissue contrast without the use of ionizing radiation or need for radioactive tracers. The results demonstrate that tumor growth inhibition following intraperitoneal chemotherapy is dependent on the dose and duration of exposure. Collectively, our results are the first to relate improved tumor control, prolonged chemotherapy tissue concentrations, and better overall survival during intraperitoneal chemotherapy that was delivered in a manner designed to precisely mimic clinical conditions.

By purposefully eliminating the variable of cytoreduction, a shortcoming of this model may be its inability to address potential synergy between surgical cytoreduction and intraperitoneal chemotherapy. Theoretically, a local inflammatory state may exist following cytoreduction that may potentiate the tumoricidal activity of HIPEC (19,20). Additionally, this mouse model mimics an open or ‘coliseum’ technique of HIPEC and may underestimate the contributions of volume, pressure, and flow parameters on drug kinetics and antitumor effect (19). In the present study, no cures were generated, and only survival prolongation was observed. All mice, regardless of treatment, required euthanasia for progressive peritoneal disease. While there may be a clinical benefit in terms of survival prolongation, the absolute impact of the various components of cytoreductive surgery/HIPEC (cytoreduction, intraperitoneal chemotherapy and hyperthermia) in patients who are ultimately cured cannot be determined from this study. It is possible that the clinical benefit from cytoreduction alone may outweigh any contribution derived from intraperitoneal chemotherapy. However, these results suggest that intraperitoneal chemotherapy, as an independent variable, may have a positive influence on clinical outcome, particularly in the background of residual microscopic disease.

The elucidation of HIPEC variables and their clinical significance in this mouse model may direct future clinical investigation and generate preclinical data as a rational basis for human clinical trials, which has been lacking. Future experiments utilizing this mouse model will interrogate the role of hyperthermia as an independent variable of HIPEC. The ability to study novel agents and immune mediated responses following HIPEC is also possible with this model. Preliminary studies from our laboratory have begun to address these issues with the ultimate goal of understanding and optimizing clinical protocols.
